# Male flowers of *Aconitum* compensate for toxic pollen with increased floral signals and rewards for pollinators

**DOI:** 10.1038/s41598-019-53355-3

**Published:** 2019-11-11

**Authors:** A.-L. Jacquemart, C. Buyens, M.-F. Hérent, J. Quetin-Leclercq, G. Lognay, T. Hance, M. Quinet

**Affiliations:** 1 Earth and Life Institute- Agronomy – Université catholique de Louvain, Croix du Sud 2, Box L7.05.14, B-1348 Louvain-la-Neuve, Belgium; 2 Louvain Drug Research Institute, Pharmacognosy Research Group - Université catholique de Louvain, Avenue E. Mounier, 72, B-1200 Brussels, Belgium; 3 Analytical Chemistry, Gembloux Agro-Bio Tech, Université de Liège, Passage des Déportés 2, B-5030 Gembloux, Belgium; 4 Earth and Life Institute – Biodiversity – Université catholique de Louvain, Croix du Sud 4, Box L7.07.04, B-1348 Louvain-la-Neuve, Belgium

**Keywords:** Evolutionary ecology, Plant ecology

## Abstract

Many plants require animal pollinators for successful reproduction; these plants provide pollinator resources in pollen and nectar (rewards) and attract pollinators by specific cues (signals). In a seeming contradiction, some plants produce toxins such as alkaloids in their pollen and nectar, protecting their resources from ineffective pollinators. We investigated signals and rewards in the toxic, protandrous bee-pollinated plant *Aconitum napellus*, hypothesizing that male-phase flower reproductive success is pollinator-limited, which should favour higher levels of signals (odours) and rewards (nectar and pollen) compared with female-phase flowers. Furthermore, we expected insect visitors to forage only for nectar, due to the toxicity of pollen. We demonstrated that male-phase flowers emitted more volatile molecules and produced higher volumes of nectar than female-phase flowers. Alkaloids in pollen functioned as chemical defences, and were more diverse and more concentrated compared to the alkaloids in nectar. Visitors actively collected little pollen for larval food but consumed more of the less-toxic nectar. Toxic pollen remaining on the bee bodies promoted pollen transfer efficiency, facilitating pollination.

## Introduction

Plant–pollinator relationships are usually considered mutualistic. Flowers offer pollen and nectar in exchange for the transfer and deposition of pollen, promoting reproductive success for the plant^1,2^. For the pollinators, pollen provides a source of proteins and lipids and nectar provides a source of carbohydrates^3^. However, pollen and nectar are costly for plants to produce. Pollen supply is limited, and pollen grains collected by bees for larval food and packaged into pollen loads are lost for plant pollination, potentially reducing plant fitness. Typical plant mechanisms for reducing pollen consumption and waste include offering alternative incentives (i.e. nectar), hiding pollen, or chemically protecting pollen^4–7^. Interest in the chemical protection of floral resources has increased, but most studies that have identified or quantified secondary defence compounds in floral rewards focus on nectar and do not consider pollen^4–8^.

Pollinators try to acquire resources at low cost. Some visitors directly access nectar without touching reproductive organs of the flower by piercing the corolla (primary robbing), using holes already present (secondary robbing) or sliding between the petals (base working)^9^. Such nectar robbing is usually observed in complex specialised flower structures, often bearing long spurs and hidden nectaries^10^. In response to this specialization, visitors with short mouthparts must make holes in floral structures or have no contact with reproductive organs, depriving plants of pollen transfer and some specialist pollinators of nectar^9–12^. Nectar robbery is not always negative for plant fitness. As nectar robbing increases the frequency of switches among inflorescences, the risk of selfing is reduced and overall higher rates of outcrossing are expected^9^. Selfing may occur in a single flower or in several flowers within the same inflorescence of a single individual; this is termed geitonogamy Selfing through geitonogamy occurs when multiple flowers are open simultaneously on a single plant^2^. Geitonogamy is linked to reduced male fitness and to inbreeding depression^2,13^. Thus, when more pollinators visit the plant, attracted by large arrays of inflorescences, but they end up probing fewer flowers because flowers are robbed, geitonogamy actually decreases and this can favour outcrossing^9,13,14^.

Many species with hermaphroditic flowers are dichogamous; that is, their pollen is ready at a different time than their stigmas. Nectar composition and volume usually vary between sexual phases in dichogamous plants^15,16^. Nectar volume, composition, and accessibility influence visitor behaviour. However, the effects of dichogamy on pollinator attractiveness and pollen transfer, and subsequent reproductive success remain unclear^2^. Showy floral displays are thought to evolve as a means to attract pollinators and thus increase mating opportunities for male-phase flowers^2,17–19^. This supposes that more pollinator visits means increased male fertility, but only a few pollinator visits will saturate female flowers^18,19^. Thus, if the number of pollinator visits limits male fitness, greater rewards and more effective pollinator signals should be favoured in male-phase flowers^19,20^. Furthermore, since male- and female-phase flowers provide pollinators with different resources (i.e. pollen and nectar, respectively), sexually dimorphic morphological, visual, or olfactory signals allow the pollinators to determine, as it were, what is ‘on the menu’, and choose their flower according to their needs for nectar or pollen^19–21^. Therefore, sexually dimorphic male- and female-phase flowers can direct pollinator movements from male to female flowers, thus making more opportunities for fertilization^20^.

**Figure 1:**
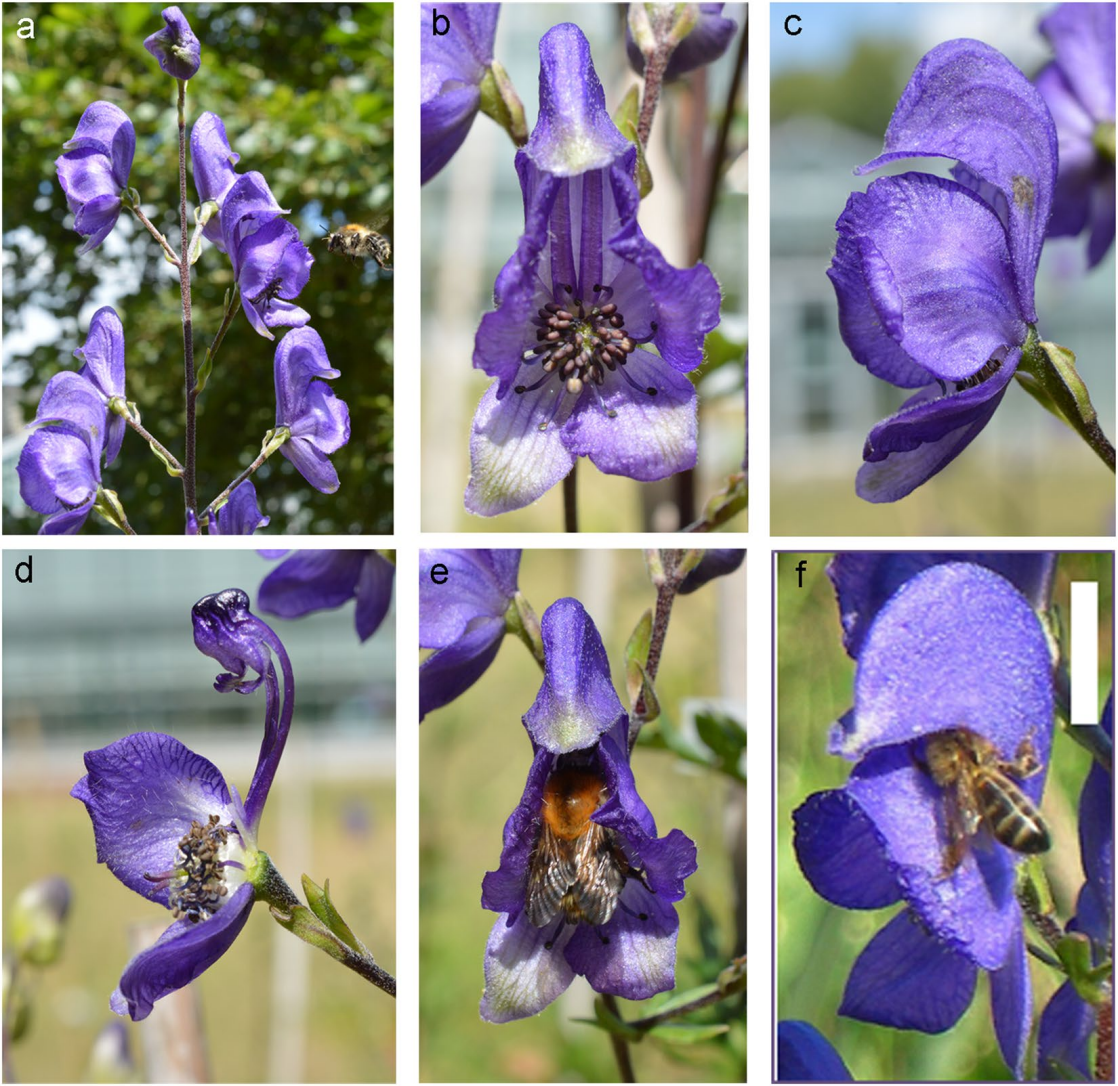
Morphology of *Aconitum napellus* spp. *lusitanicum*. Inflorescence (**a**), front view of a flower showing anthers and stigmas (**b**), side view of a flower (**c**), cut open flower showing the two nectaries at the end of the spurs (**d**). Legitimate visit by *Bombus pascuorum* (**e**). Illegitimate visit (base working) by *Apis mellifera* (**f**, white bar = 1 cm).

Studies that examine the balance between pollinator attraction (signals) and rewards as a function of dichogamous sexual phase are rare, as are studies that focus on male reproductive success in toxic plant species. Here, we examined reproductive fitness in male- and female-phase flowers in the protandrous hermaphrodite *Aconitum napellus* spp. *lusitanicum* Rouy (Common monkshood, Ranunculaceae, Fig. 1). In contrast to previous studies that examined only one aspect of plant–pollinator interactions (toxicity, robbing, signals, etc.), we combined observations in natural populations (pollinator behaviour) with experimental approaches (detection of toxic compounds) and fine chemical analyses of signals and rewards.

*Aconitum* spp. are bee-pollinated plants^4,8,9^. Inflorescences of *A. napellus* spp. *lusitanicum* are composed of numerous (up to 50) large and zygomorphic flowers (Fig. 1a–c)^21^. The flowers are strongly protandrous and exhibit a longer male phase (5–6 days) than a female phase (1–2 days)^9^, similar to other *Aconitum* species^22–24^. In each inflorescence, the flowers at the bottom open first; these female-phase flowers open before the younger male-phase flowers at the top of the inflorescence.

Bees usually begin to forage at the bottom of the inflorescence and proceed toward the top^11^. Due to the longer male phase, floral display is male-biased^9^. The upper, helmet-shaped petaliferous sepal protects two spurred nectariferous petals (Fig. 1d); as they crawl into the helmet, pollinators go over the stigma and the anthers^9,11^. Most of the pollinators visiting flowers are bumblebees (up to 95% of all insect visitors) and honeybees (18–54%), but the number of each kind of visitors changes in different years and locations^8,9,25^. Insects make two types of visits to *A. napellus* flowers: ‘legitimate’ visits in which the visitor contacts the reproductive organs allowing the pollination of the flower, and ‘illegitimate’ visits in which the insect robs the nectar and does not participate to pollination (Fig. 1e,f)^9,11^. Long-tongued bumblebees, *Bombus hortorum* (tongue length of about 12 mm) and *B. pascuorum* (tongue length of about 8 mm), are the main pollinators, whereas short-tongued (tongue length of about 6 mm) bumblebees (*Bombus terrestris* and *B. lucorum*) and honeybees (*Apis mellifera*) are mainly considered robbers of nectar^8,9,25^. The proportion of robbed flowers varies from 2 to 23% according to the population and the year^9,25^. Fruit set in natural populations remains high (90–93%), showing that female-phase flowers continue to attract pollinators despite nectar robbery^9^.

As with nearly all species in the Ranunculaceae, *A. napellus* is poisonous. All *Aconitum* species contain diterpenoid aconitine-like alkaloids; these toxic alkaloids affect the neural and cardiovascular systems in insects and mammals^26,27^. The main alkaloids in *Aconitum* plants are aconitine and its *N*-methyl homolog mesaconitine^26^, although variations in composition and concentration have been observed among species and in different parts of the same plant^4,8^.

In this study, we compared floral signals (colour, size, and odour) and floral rewards (nectar and pollen quantity, composition, and toxicity) between male- and female-phase flowers. We hypothesized (i) that male reproductive success is more pollinator-limited than female reproductive success and that floral signals and rewards should be enhanced in male-phase flowers as a result and (ii) that at the insect level, visitors should prefer to forage flowers only for nectar due to the toxicity of the pollen.

We addressed the following questions. (i) Do floral signals (size, shape, and odour) differ between male and female phases? (ii) Do floral reward quantities and compositions differ between male and female phases? (iii) Do pollen and nectar rewards differ in total and profile of alkaloids? (iv) Do insect visitors detect alkaloid toxicity? (v) Do insect visitors differ in their behaviour and pollination efficiency?

## Results

### Do floral signals differ between male and female phases

**Figure 2:**
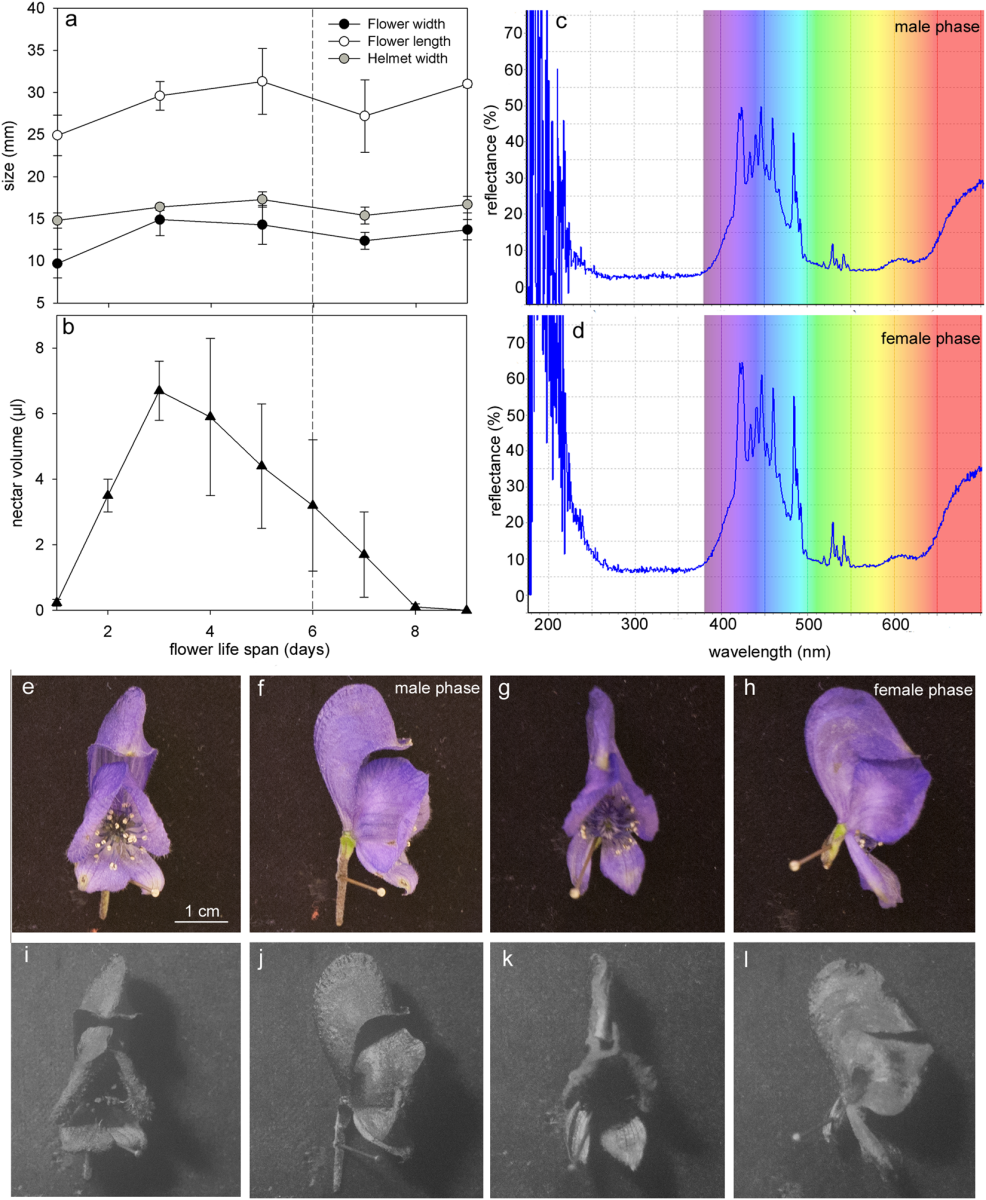
Floral traits and nectar production during male and female phases of *Aconitum napellus*. Variations of floral traits (**a**) and nectar volume (**b**) during flower life span. Tepal color (**c**,**d**) and flower UV reflectance (**e**–**i**) at male (**c**,**e**,**f**,**i**,**j**) and female (**d**,**g**,**h**,**k**,**l**) phases. Dashed line separates male and female phase in (**a**). Photographs in both visible (**e**–**h**) and UV light (**i**–**l**) for analysis of flower UV reflectance. Male phase corresponds to flowers from 1 to 6 days after anthesis, and female phase corresponds to flowers from 7 to 9 days old. (N = 24 flowers per day).

**Figure 3:**
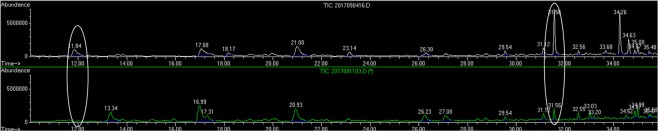
Total ion chromatograms (same scale) of VOCs from *Aconitum napellus* flowers showing the occurrence of *trans*-β-ocimene (Retention time 11.84 min) and benzene ethanol (Retention time 31.56 min). Distinction can be made between male flowers (upper trace) and female (bottom trace) where the two compounds are strongly less represented (*trans-*β-ocimene was detected in traces).

Male-phase flowers produced a mean of 0.83 mg of pollen, from 40 to 53 stamens, corresponding to a minimum of 180,000 pollen grains per flower. Some pollen grains were still present during the female phase. Flower morphology did not significantly differ between male and female phases (Table 1, Fig. 2a). Tepal colours (spectrometry) and UV reflectance also did not differ (Fig. 2c–l). Fragrance analyses revealed complex chromatographic profiles with many well-resolved peaks (Fig. 3). Benzene ethanol and *trans*-β-ocimene (122 ± 112 and 104 ± 41 ng h^−1^, respectively) were detected within the volatile organic compounds (VOCs) from male-phase flowers but were found only in minute amounts (benzene ethanol, 13 ± 13 ng h^−1^, F_1,6_ = 3.7382, *P* = 0.1014) or not at all (*trans*-β-ocimene < limit of detection [LOD], F_1,6_ = 25.73, *P* = 0.0023) in the female-phase flowers.

**Table 1:** Flower morphology, and nectar resources in the male- and female-phase flowers (N = total number of samples).

Parameters	N	Male	Female	Wilcoxon Test
Corolla length	26	2.89 ± 0.37	3.25 ± 0.30	*Z* = 1.212; *p* = 0.225
Corolla diameter	26	1.23 ± 0.32	1.1 ± 0.12	*Z* = −0.185; *p* = 0.854
Helmet width	26	1.56 ± 0.11	1.60 ± 0.11	*Z* = −0.449; *p* = 0.653
Nectar volume (µL flower^−1^)^*^	24	20.7 ± 5.3	4.8 ± 5.3	*Z* = 4.943; *p* < 0.0001
Total sugar concentration (µg mg^−1^)	6	444.5 ± 36.8	420.7 ± 38.9	*Z* = 0.436; *p* = 0.662
Sucrose (%)	6	82.8 ± 1.7	83.2 ± 1.8	*Z* = −0.873; *p* = 0.383
Fructose (%)	6	17.4 ± 1.5	16.6 ± 1.9	*Z* = 0.886; *p* = 0.376
Glucose (%)	6	0.4 ± 0.2	0.2 ± 0.0	*Z* = 1.291; *p* = 0.197

^*^Sum of the daily nectar production during male and female phases.

### Do floral resources differ between floral phases

Nectar volumes significantly differed between male and female phases, with nectar production peaking on the third or fourth day after anthesis and decreasing continuously thereafter (Fig. 2b). As a result, flowers produced more nectar during the male phase than during the female phase (Table 1). The total sugar concentration and the proportions of sugars did not significantly differ between male and female phases. Sucrose largely dominated the nectar composition (around 80%) with sucrose/hexose ratios higher than 4.

### Do pollen and nectar differ in total alkaloids and profiles

Liquid chromatography-mass spectrometry (LC/MS) analysis revealed that the pollen and nectar contained unique alkaloid profiles (Table 2). Pollen had more diverse alkaloids than nectar: we analysed 37 different alkaloids and identified 22 in pollen but only 4 in nectar. Aconitine was one of the major alkaloids found in pollen (Table 2). Aconitine concentration in pollen was 232 µg g^−1^ and the concentration in nectar was lower than 0.005 µg·g^−1^. In pollen, the main three alkaloids were aconitine, 6-*O*-acetylacosepticine, and napelline. The concentration of 6-*O*-acetylacosepticine and napelline was about 7 to 9% of the concentration of aconitine. The other detected alkaloids represented less than 6% of the content of aconitine. Nectar contained only three alkaloids among those present in pollen (Table 2). Concentrations of aconitine and napelline were similar in nectar. Concentrations of 6-demethyldelphatine and 18-demethylpubescenine (not detected in pollen) were about 2 and 16 times lower than aconitine contents, respectively.

**Table 2:** Alkaloid compounds detected in pollen and nectar.

Molecular formula	m/z	Rt/min	Assignment*	Pollen (%)	Nectar (%)
C_34_H_47_NO_11_	646	8.7	Aconitine	100	100
C_33_H_45_NO_10_	616	8.4	Hypaconitine	0.4	nd
C_22_H_33_NO_3_	360	2.2	Napelline A	6.7	102
C_22_H_33_NO_3_	360	5.4	Napelline B	8.5	nd
C_24_H_39_NO_6_	438	2.2	Neoline A	2.0	nd
C_24_H_39_NO_6_	438	5.9	Neoline B	4.5	nd
C_33_H_45_NO_11_	632	8.3	Mesaconitine	0.3	nd
C_25_H_41_NO_9_	500	2.2	Aconine A	0.3	nd
C_25_H_41_NO_9_	500	5.4	Aconine B	0.3	nd
C_31_H_43_NO_10_	604	8.0	Benzoylmesaconine	0.3	nd
C_31_H_43_NO_9_	574	7.8	Benzoylhypaconitine	1.8	nd
C_23_H_37_NO_6_	423	2.2	Acosepticine A	0.4	nd
C_23_H_37_NO_6_	423	5.4	Acosepticine B	0.3	nd
C_25_H_39_NO_7_	465	6.6	6-O-Acetylacosepticine A	8.8	nd
C_25_H_41_NO_7_	467	2.2	6-Demethyldephatine A	4.4	nd
C_25_H_41_NO_7_	467	5.7	6-Demethyldephatine B	5.3	nd
C_25_H_41_NO_7_	467	6.0	6-Demethyldephatine C	5.7	56.2
C_25_H_39_NO_8_	481	8.4	18-Demethylpubescenine	nd	6.0
C_27_H_43_NO_8_	509	6.9	Leucostine	5.2	nd
C_32_H_44_N_2_O_7_	568	9.0	Acoseptine	3.6	nd
C_32_H_44_N_2_O_9_	600	8.2	N-Acetylsepaconitine A	0.2	nd
C_32_H_44_N_2_O_9_	600	8.6	N-Acetylsepaconitine A	0.5	nd

*m/z* = mass charge ratio. RT = Retention time. N = 3. Assignment based on MS data, fragmentation and presence in other *Aconitum* species. The content of identified alkaloids is expressed as a relative value compared to aconitine (232 µg g^−1^ in pollen, 5 10^−3^ µg g^−1^ in nectar, considered as 100%). nd = not detected.

### Do insect visitors detect alkaloid toxicity

**Figure 4:**
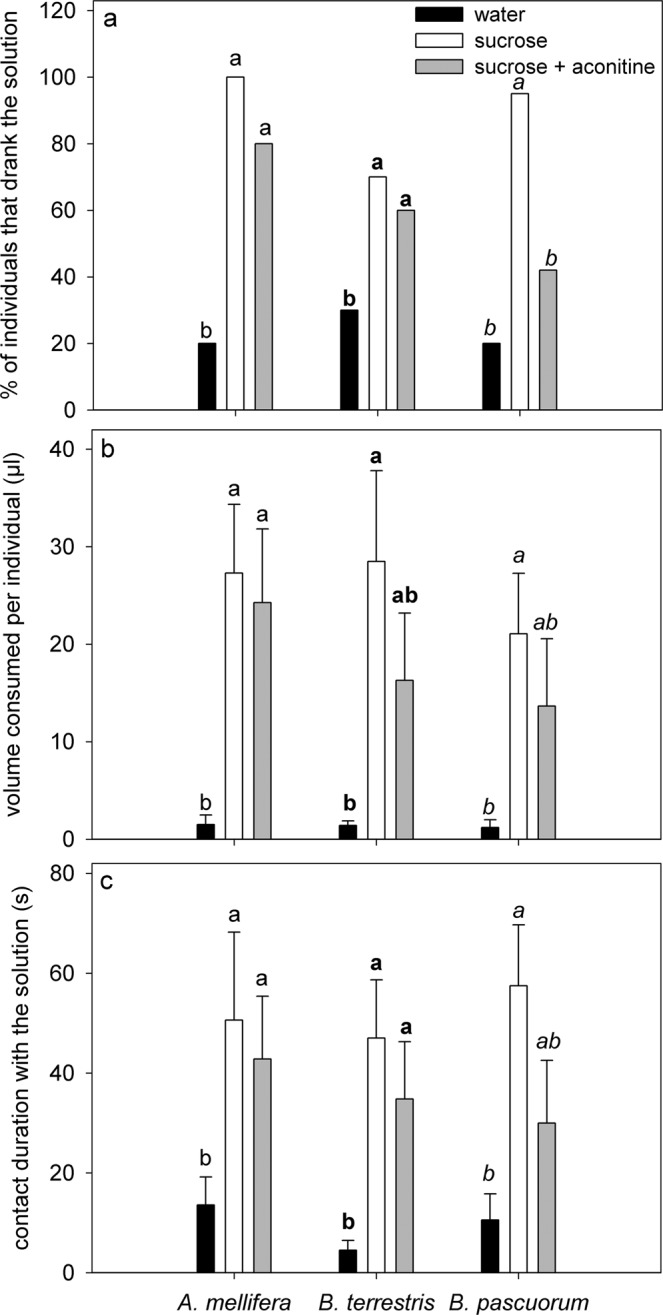
Detection of aconitine by the main bee visitors (*A. mellifera*, *B. hortorum*, and *B. pascuorum*) of *Aconitum napellus*. Percentages of individuals that drank the solution (**a**), total volume (µL) consumed per individual (**b**), and contact duration (sec) with the solution (**c**) (N = 10 per insect species and per solution). For each insect species, solution type followed by different letters are significantly different at *P* < 0.05.

Most *B. pascuorum* individuals tested rejected artificial solutions with aconitine (Fig. 4a). All together, the percentage of bees that consumed sucrose solution was significantly higher than the percentage that consumed sucrose + aconitine solution (*X*^2^ = 10.806, *df* = 1; *P* = 0.001). Consumption rates and contact time with aconitine-laced solutions, when consumed, were slightly lower than for the pure sucrose solution mainly for *B. pascuorum* (Fig. 4b,c). Long-tongued *B. pascuorum* individuals were more deterred by aconitine than individuals from short-tongued species (*A. mellifera* and *B. terrestris*); the combined deterrence index reached 0.40 for *A. mellifera*, 0.64 for *B. terrestris*, and 0.80 for *B. pascuorum*.

### Do insect visitors differ in their behaviour and pollination efficiency

In natural populations, the main insect visitors differed in their visitation behaviour (Table 3). *A. mellifera* individuals were abundant visitors with 77 individuals out of 183 observed visitors and only visited few flowers per inflorescence (total of 291 visited flowers) for nectar (legitimately (24%) or by base working robbery, sliding between the tepals (76%)) whatever the sexual phase of visited flowers (always more male phase flowers in all populations and years). Short-tongued *B. terrestris* individuals also only visited flowers for nectar (only three individuals, legitimately or illegitimately by piercing a hole in the upper tepal). *B. hortorum* and *B. pascuorum* individuals (long-tongued, total of 102 individuals out of 183 visitors) typically made legitimate visits (99%) to many flowers per plant (total of 1065 visited flowers). Nevertheless, they collected much less pollen (1%) than nectar (99%). Pollen was rarely actively collected and packaged in the corbiculae (pollen loads). Since insects rarely visited flowers for pollen collection, we obtained only 23 pollen loads from *B. hortorum* (all years and populations combined) and 2 from *B. pascuorum*. These pollen loads contained relatively few pollen grains (mean 370 ± 115 per load). *A. napellus* pollen represented 56.3 ± 12.6% of the pollen collected.

**Table 3:** Behaviour and resource collection for the three main visitors (*Apis mellifera, Bombus hortorum*, and *B. pascuorum*) in the four populations in South Belgium (total of 800 minutes of observations, 2011 and 2012 combined). N = number of visitors. Different letters indicate significant differences between data. Data are means ± SD.

Visitor	N	Numbers of visited flowers			
		Total/plant	Pollen visited	Nectar visited	Nectar robbed
*A. mellifera*	78	3.7 ± 3.1^b^	0	0.9 ± 1.3^b^	2.8 ± 3.50^a^
*B. hortorum*	60	12.0 ± 9.6^a^	0.2 ± 1.2	11.7 ± 9.5^a^	0.1 ± 0.3^b^
*B. pascuorum*	26	8.8 ± 9.8^ab^	0.1 ± 0.3	8.7 ± 9.9^a^	0^b^
		*X*^2^ = 18.765; *p* < 0.0001	*X*^2^ = 1.165; *p* = 0.5585	*X*^2^ = 35.056; *p* < 0.0001	*X*^2^ = 40.384; *p* < 0.0001

**Figure 5:**
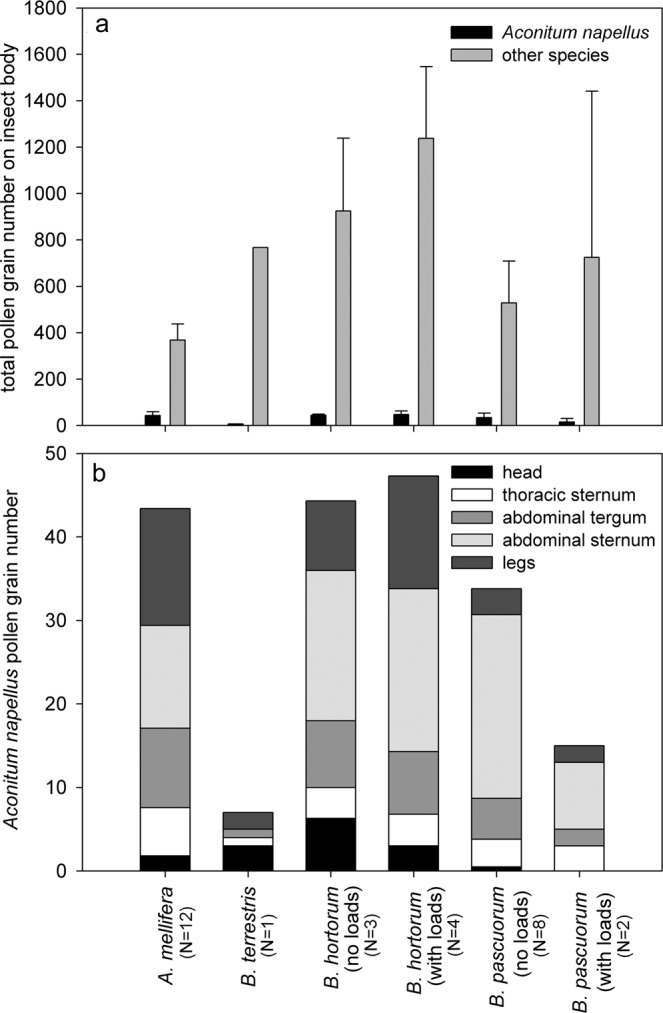
Pollen grains transported by the different bee visitors caught in natural populations. Pollen grains of *Aconitum napellus* and other species on the body of the bee visitors caught (**a**), number and distribution of pollen grains of *Aconitum napellus* on the different body parts (**b**).

Most insects had *A. napellus* pollen grains on their bodies (Fig. 5). Efficient pollination requires that pollen be present mainly on the lower parts of the visitor’s body (thoracic and abdominal sternum) to promote contacts with stigmas. With the exception of one *B. terrestris* individual, all other insects had aconite pollen on their bodies, available for pollinating flowers. Honeybees that legitimately visited flowers transported similar quantities of aconite pollen to that of the long-tongued *B. hortorum*.

## Discussion

Our results revealed a clear sexual difference in floral scent production, with one alcohol, benzene ethanol, and one monoterpene, *trans*-β-ocimene, produced exclusively or at higher rates in male- than in female-phase flowers. It is the first time than odour detection was tried on *Aconitum* species. Odours specific to male-phase flowers related to pollen or anther odours have been reported in other species^28^. The two particular components we identified have also been detected in other floral scent compositions^21,29–31^ with β-ocimene detected in 71% and benzene ethanol in 54% of the 991 studied species^32^. In dioecious species, more volatiles have been detected in male flowers compared to female flowers^18–21,30,33^. However, most (62%) of species reviewed by Ashman^18^ presented a similar composition of volatiles in both floral sexes. Nevertheless, we were not able to confirm the attractiveness of benzene-ethanol for the three insect visitor species tested using an olfactometer, as individuals did not preferentially choose the odour-marked channel^34^.

Nectar production differed between the floral sexual phases in our species, with 4.3-fold more nectar produced during the male phase than the female phase. Male-biased nectar production was also documented in *Aconitum carmichaelii*, whereas nectar production was female-biased in other protandrous species^15,22,35–37^. Male-biased nectar production is common in hermaphrodites, suggesting that this secretion pattern has evolved to ensure more visits in male-phase flowers^15,22,38,39^.

Floral sexual phase had no impact on the total sugar concentrations or on the relative proportions of sugars. The nectar of *A. napellus* ssp. *lusitanicum* predominantly contains sucrose (83%). Sucrose-rich or sucrose-dominant nectars with high total sugar concentrations have been detected in other long-tongued bee-pollinated *Aconitum* species^16^. The prevalence of sucrose is a common characteristic of nectars hidden within deep flowers^16,20^.

Such high-quality nectar in large volumes should attract bees^39^. The bottom of the inflorescence has more female flowers and thus more nectar compared with the top of the inflorescence. This change in nectar availability may improve plant fitness^20,39^, as pollinatorys may avoid the female-phase flowers and concentrate on the more-rewarding male-phase flowers. This may benefit the plant because in protandrous species, more ovules become available over the flowering season, as less pollen is available^16,40^. With a male-biased display of flowers, *A. napellus* should preferentially attract pollinators that disperse pollen grains and contribute to male fitness^13,23^. Deposition of pollen on female-phase flowers is maintained by the pattern of upward foraging commonly exhibited in bumblebees on vertical inflorescences^14,23,36,41^.

We detected 37 different diterpenoid alkaloids in the floral resources of *A. napellus*, with more diverse profiles and higher concentrations in pollen than in nectar. Total concentration (more than 200 µg g^−1^) in pollen was in the same range as in the roots (A.-L. Gauthier, unpublished data). Similar alkaloids have been detected in other *Aconitum* species^8,26,27,42^. Pollen (around 4 10^−3^ µg g^−1^) also had significantly higher concentrations of alkaloids than leaves or flowers, and nectar had the lowest concentration of alkaloids in *A. septentrionale*^4^. In the few published comparisons of alkaloid levels between nectar and pollen, concentrations in nectar tended to be lower than in pollen, with concentrations in pollen equal to or exceeding that of other organs such as leaves^43^.

Measuring the toxicity of *A. napellus* floral rewards may require measuring the survival of growing larvae or individual bees. However, we can estimate that toxicity level is high based on our results and published data. First, we found that *A. napellus* ssp. *lusitanicum* had a high concentration of aconitine-like alkaloids in pollen (232 µg g^−1^); this exceeds the ED50 of all the alkaloids that Detzel and Wink tested^44^ for *A. mellifera*. Second, the major alkaloid in pollen was the highly toxic alkaloid aconitine^26,27^. Alkaloid concentrations in pollen of *A. napellus* are thus potentially lethal or sublethal^45^.

Our gustatory tests demonstrated that some bee species were more sensitive to alkaloids than others. Nectar-robbing short-tongued bees (*B. terrestris* and *A. mellifera*) were less deterred by the same concentrations of aconitine than *B. pascuorum*. Indeed, *B. terrestris* has higher sensitivity to alkaloids compared with other plant compounds and for most compounds, these bees can only taste compounds present at greater than 10 ppm^45–47^. Low levels of toxic alkaloids in nectar do not affect bumblebee foraging behaviour^46^. Detection thresholds for other *Bombus* species have not been studied. Indeed, extrapolating our experimental data to field conditions, insect visits ceased at alkaloid concentrations up to 200 µg g^−1^, contrary to the results of Barlow *et al*.^8^. Bees act as if they were balancing economic gains (sugar collection) against toxicity^48^. For example, Barlow *et al*.^8^ estimated the cost of nectar robbery by calculating the nectar that a pollinator could get from robbed flowers in *A. napellus*. They multiplied the percentage of flowers with nectar (mean of 48) by 26.6 ATP for each flower, giving a total pay-off of 12.7 10^19^ ATP per visit or 950 10^19^ ATP per population.

Pollen toxicity may be considered a chemical defence that helps plants decrease herbivory and excessive harvesting of pollen^43,44^. As alkaloids at high concentrations could deter, harm, or kill visitors^44,49^, they may deter non-pollinating insects; this may prevent pollen from being wasted and thus improve pollen transfer among plants^43,50^. This protective role has been proposed for different genera in the Ranunculaceae^4,51,52^. Pollen collected for larvae food and packaged in corbicula is lost for pollination. When insects detect the toxicity of pollen and do not actively collect pollen or dislike grooming, they leave more pollen grains on the body parts that make contact with stigmas, thus ensuring efficient pollination^52^. All the observed visitors carried *Aconitum* pollen on all parts of their bodies, confirming their effective role in pollination.

In contrast to its pollen, *A. napellus* nectar has a low concentration of alkaloids and should be suitable for a large range of foragers^4,44^. Insect species mainly visited *A. napellus* for nectar^12,24^. Moreover, several individuals from species considered as usual robbers were observed legitimately visiting flowers. The legitimate visits to female-phase flowers likely would be enough to ensure the observed high pollination rate and high fruit set (90–93%).

Both mechanisms, the deterrence effect of pollen toxicity and high production of sugar-rich nectar work together to ensure the reproductive success of this specialised protandrous plant species, mainly by high male fitness.

## Methods

### Plant material

Observations and chemical analyses were conducted from 2011 to 2018 on a collection of plants grown in the experimental garden at the University Louvain-la-Neuve campus (50°39′55″N; 4°37′11″E). Before and during their blooming period (July–August) plants were placed in the University’s growth chambers under controlled conditions (24/22 °C; 16 L:8D, RH 80 ± 10%).

### Insect material

The main insect visitor species of *A. napellus* (*A. mellifera*, *B. pascuorum*, and *B. terrestris*) were used in trials of flower attractiveness and gustatory preference. *A. mellifera* and *B. pascuorum* individuals (10 per test and treatment) were collected in the wild around the university campus (minimum of four locations per species) in the morning prior to tests. As *B. terrestris* individuals cannot be easily distinguished in the field (similar morphology for several species), we used bumblebees from four hives (Biobest, Westerloo, Belgium) placed in the vicinity of the university buildings to obtain individuals for experimentation.

### Floral traits

#### Pollen counts

The anthers of 10 flowers at male and female phases were collected, counted, dried and placed on a sieve (90 µm) to be rubbed to collect pollen grains. The quantity of pollen grains was weighed. Pollen counts were performed at Lille-1 University (Evo-Eco-Paleo Unit). We used a particle counter [CASY, model TT (Innovatis, Bielefeld, Germany)] to estimate the number of pollen grains in these samples. The day before pollen counts, samples were placed in a tube heater until most of the ethanol evaporated. We then forced anther dehiscence by placing samples in an oven at 56 °C overnight. We added 1 mL of distilled water to each pollen sample. Tubes were then sonicated and vortexed to separate pollen grains from the anthers and each other. Pollen solution (200 µL) was then diluted in 5 mL of isotonic measurement buffer (CASY® ton). The particle counter analysed three 400-µL samples from the pollen suspension, providing total particle count in this 1200 µL volume, as well as counts for 400 size classes ranging from 0.125 to 150 µm. Comparisons with blank samples allowed us to identify peaks of particles, with size ranging from 20 to 30 µm, which corresponds to pollen grains. The number of pollen grains per anther was estimated by multiplying the values provided by the particle counter by the dilution ratio (i.e. (1000/200) × (5000 + 200)/1200).

#### Flower colour and morphology

We measured floral traits (corolla length, corolla diameter, floral colour, and UV reflectance) on 10 flowers per phase (from 10 different individuals). Helmet width, corolla length and diameter were measured with a calliper. We measured tepal colour reflectance spectrum using a fibre optic spectrometer (Avaspec-ULS2048) and a xenon-pulsed light source (AvaLight-XE, Avantes, Apeldoorn, The Netherlands). For UV reflectance measurements, flowers were fixed on a black background and photographed with a Nikon D40 (Coastal Optical 60 mm 1:4 UV-VIS-IR ApoMacro; Filtre X-Nite 330).

### Floral volatile collection

We sampled volatile organic compounds (VOCs) from intact male- and female-phase flowers (N = 4 for each phase). Each flower was individually inserted in a glass bulb (50-mm length and 30-mm diameter), with a 17-mm-diameter opening. To prevent air contamination, one Teflon tube filled with active charcoal was connected to one end of the glass bulb and two glass Tenax TA tubes (6 mm × 4 mm, 7 inches; 60–80 mesh, Gerstel, Sigma-Aldrich, Overijse, Belgium) were connected to the other end. The Tenax tubes were connected to calibrated air pumps (Gilair plus, Sensidyn, St. Petersburg, USA) at a flow rate of 50 mL·min^−1^ for 180 min (between 11:00 and 14:00 h). Lab conditions ranged between 21.5 and 23.5 °C with 45 to 55% RH.

Trapped VOCs were then thermally desorbed on an Agilent 6800 gas chromatograph fitted with a Gerstel TDU thermodesorption unit and CIS cryocooler maintained at −50 °C. Immediately after thermodesorption, the CIS unit T° was programmed from −50 °C to 300 °C at 12 °C sec^−1^. The GLC apparatus was coupled to an Agilent 7975 C mass spectrometer. Analytical conditions were as follows: split mode injection (with split ratio of 50:1, helium at 1.6 mL min^−1^ as carrier gas), VF-Max MS 30 m x 250 µm (df = 0.25 µm) column (Agilent). The temperature programme allowing the best separation (preliminary tests not shown) was fixed as follows: 40 °C (5 min) then to 75 °C at 4 °C min^−1^ and to 115 °C (3 °C min^−1^) and finally to 250 °C at 13 °C min^−1^. MS conditions were as follows: EI mode at 70 eV, source T° = 250 °C, MS quadrupole at 200 °C, and scanned mass range 35 to 500 amu.

The recorded chromatograms were systematically interpreted to search for VOCs specific to male or female phases. When detected, the molecules were identified according to computed databases (Wiley 250.L and PAL 600). The identifications were corroborated based on the retention data of pure molecules commercially available. They were also quantified relative to highly pure internal standard (limonene, 1 µL methanol solution at 0.67 µg µL^1^ automatically added to each desorption tube before the thermal process).

Ambient air was also collected as a control to identify background contaminants. To prevent a possible breakthrough, a second glass cartridge loaded with the same adsorbent (Tenax TA) was systematically placed in tandem. The analysis did not reveal any traces of the molecules of interest.

### Alkaloid collection and analyses

Anthers were collected from 50 flowers (from 10 individuals), dried and then crushed on a sieve (90 µm) to collect pollen grains. Three replicates of 15 mg of pollen were sampled for alkaloids. Samples were frozen (−80 °C) for 2 h and then freeze-dried. Dry samples were ground to a fine powder with liquid nitrogen, and weighed amounts of powder were placed in a 1.5-mL microcentrifuge tube (Eppendorf, Hamburg, Germany). Alkaloids were extracted using a tissue homogenizer (VWR Ultrasonic Cleaner) for 10 min in 100 µL of extraction buffer (70% aqueous methanol and 0.5% formic acid). Following centrifugation at 12,000 rpm for 5 min (Centrifuge 5430 R), the supernatant was transferred to a 1.5-mL microcentrifuge tube. After drying in a SpeedVac, alkaloids were resuspended in 200 µL of LC-MS-grade methanol and filtered with a 0.45-µm PTFE syringe filter (Whatman).

Nectar was collected in 5-μL glass capillary tubes (Hirschmann Laborgeräte, Eberstadt, Germany) from 50 flowers (from 10 individuals). Three replicates of 15 µL of nectar were dried in a SpeedVac and resuspended in 200 µL of LC-MS-grade methanol before filtration with a 0.45-µm PTFE syringe filter (Whatman).

Chemical analyses were performed using an LC-MS/MS system consisting of a Thermo Accela pump, autosampler, photodiode array detector, and a Thermo Scientific LTQ orbitrap XL mass detector (MASSMET, Louvain Drug Research Institute, Woluwe, Belgium). A Phenomenex Gemini C18 column was used (2 mm × 150 mm, packed with 3-µm particles). A binary solvent system with a flow rate of 300 µL min^−1^ was used: solvent A, HPLC-grade water (0.1% formic acid); and solvent B, LC-MS-grade methanol (0 min: 95% A, 10–12 min: 0% A, 13–17 min: 95% A). A 10-µL volume was injected and the column temperature was set at 30 °C. High-resolution MS was performed with an electrospray ionisation source in positive mode. The following inlet conditions were applied: capillary temperature, 275 °C; capillary voltage, 13 V; tube lens, 140 V; sheath gas flow, 30 a.u.; and auxiliary gas flow, 10 u.a. Data acquisition and processing were performed with Xcalibur software. This method covered the mass range of all alkaloids (MW from 329 to 673). Alkaloids from pollen and nectar were detected and tentatively identified by high-resolution MS. Fragmentation obtained by collision-induced dissociation helped in the identification. Alkaloid concentrations were calculated based on an aconitine standard curve in MeOH and expressed as “aconitine-equivalent”. Analysed alkaloids were selected based on their presence in other *Aconitum* species^4,8^.

### Nectar production and sugar composition

Nectar was collected in 5-μL glass capillary tubes (Hirschmann Laborgerate, Eberstadt, Germany) from flowers of the plants transferred in the growth chambers at the University campus (no insect visits). We estimated the nectar production trend during the day at the beginning of the anthesis by sampling 2 flowers from 12 individuals every 2 hours during 10 hours. We also measured the total nectar production per flower (2 flowers from 12 individuals) for the same flowers every 2 days during the entire (9 days) flower life span, at the same time each day, 2 hours after the light was switched on. Total production for male and female phases was estimated from sampling at the end of the male (5 days after anthesis) and female (8 days) phases.

Nectar samples were stored at −80 °C until chemical analyses were performed. After derivation, sugar identification and quantification were performed by GC-MS (Thermo Trace 1310 /Thermo ISQ-QD). A Restek Rxi-5Sil MS (30 m x 0.25 mm ID, 0.25 µm *df*) column was used. The flow rate was 1.0 mL min^−1^ (helium). The split ratios injected were 1/10 for the hexoses and 1/100 for sucrose. The following inlet conditions were applied: oven temperature, 105 °C for 4 min, then to 280 °C at 15 °C min^−1^ (for 20 min) and injector temperature of 250 °C.The total sugar content of nectar per flower (mg) was then calculated as volume of nectar (µL) x total sugar concentration (mg/µL).

### Insect behaviour

In 2011 and 2012, investigations took place in the four remaining natural populations in fens in South Belgium. Of these, the nature reserve “Les Abattis” (49°40′50″N; 5°32′59″E) contains the largest and densest population (2970 m^2^ with 1330 ± 1030 *A. napellus* flowers m^−2^). At “Fouches” (49°4′8″N; 5°43′06″E), which has the second largest population (1788 m^2^), individuals of *A. napellus* were very scattered over the area and at much lower densities (176 ± 177 flowers m^−2^) “Chantemelle’ (49°39′37″N; 5°39′37″E)’ had a discontinuous population consisting of three patches (161, 423, and 500 m^2^) with relatively low plant and flower densities (179 ± 125 flowers m^−2^). “Sainte-Marie” (49°40′06″N; 5°32′56″E) had a tiny and patchy population (292 m^2^, 152 ± 163 flowers m^−2^) stretching about 150 m along a former railway track.

Flower visitors were recorded in 10-min observations during peak flowering on three consecutive days in 2011 (between August 30 and September 3) and on four days in 2012 (between August 21 and 27) with dry and warm weather conditions. The proportion of male phase flowers, during these days and in all populations, was higher than female phase flowers (about 85%). Flowers within 1 m^2^ (or about five plants) were observed at the same time. A minimum of ten observations per population were conducted (maximum 16), spread over the entire populations and over different times during the day. For each visitor, we recorded the species, number of robbed and unrobbed flowers visited per plant and patch, time spent per single flower as well as their foraging behaviour (pollen or nectar collection, legitimate or illegitimate visit, referred to as robbing (base-working, primary and secondary robbing). During 530 min of flower observations in 2011 and 400 min in 2012, we recorded 183 flower visitors. Bumblebees (*B. pascuorum* and *B. hortorum*) and honeybees (*Apis mellifera*) were the major flower visitors; only 3 *B. terrestris* individuals were observed.

### Pollen carrying capacity

Thirty individuals per species were killed with ethyl acetate, and individually stocked. Pollen was removed from the different insect body parts with small cubes of gelatine-glycerine^53^. Pollen grains concentrated in the corbicula were removed and not included in analyses. All the pollen grains were counted by light microscopy.

### Pollen collection purity

We sampled pollen loads from bumblebees visiting *A. napellus* in the natural populations. In the lab, pollen loads were acetolysed^53^. At least 400 randomly chosen pollen grains of each of the 25 pollen loads were identified and counted under light microscopy.

### Gustatory responses of insect visitors

We followed the protocol of Ma *et al*.^54^ for aconitine detection. When caught, individual bees were starved for 3 hours in a plastic vial (7 cm long, 2.8 cm inner diameter) at room temperature and in complete darkness in the lab. We transferred each bee to a holding tube, a 15-mL centrifuge tube with a 4-mm hole drilled at the tip and a piece of steel mesh fixed inside. After a pre-test phase with a droplet of sucrose, we presented the test solution in a 100-µL glass capillary tube and gently squeezed the tubing to maintain the solution at the tip of the tube. Any insect with no proboscis extension after 5 minutes was removed from the test. Test phases were 2 minutes long, and volume of the solution before and after the test were recorded with a calliper.

Tests were performed with three visitor species (*A. mellifera*, *B. pascuorum*, and *B. terrestris*). Ten individuals per insect species were tested with each solution. Sucrose (430 µg mg^−1^) and aconitine (232 µg g^−1^) concentrations corresponded to those found in our species. Three solutions were presented: deionized water alone (control), pure sucrose solution, and sucrose plus aconitine solution.

For all tests, a deterrence index was calculated for each subject from the choice to drink, the volume of solution consumed, and the time of contact with the solution as follows: ID = 1- (choice for sucrose/choice for sucrose + aconitine) × (volume consumed of sucrose/volume consumed of sucrose + aconitine) × (time of contact with sucrose solution/time of contact with sucrose + aconitine solution).

### Statistical analyses

The normality of the data was estimated using Shapiro–Wilk tests, and homoscedasticity was verified using Levene’s tests. When normality and homoscedasticity requirements were fulfilled, analyses of variance (ANOVAs) were performed (floral phase effect on floral volatiles). Otherwise, nonparametric tests were performed using Wilcoxon test for comparison of two conditions (floral phase effect on flower morphology and nectar parameters) and Kruskall-Wallis test for comparison of more than two conditions (insect and solution effect on consumed volume and contact duration with the solution in gustatory experiments, insect effect on the visitation behaviour) and chi-square tests were performed to compare the percentages of individuals in gustatory experiments. ANOVA and non parametric analyses were followed by multi pairwise comparison when more than two conditions were compared. All analyses were conducted in SAS Enterprise Guide 7.1. The data are presented as means ± standard deviations.
